# Regulation of taurine transport at the blood-placental barrier by calcium ion, PKC activator and oxidative stress conditions

**DOI:** 10.1186/1423-0127-17-S1-S37

**Published:** 2010-08-24

**Authors:** Na-Young Lee, Young-Sook Kang

**Affiliations:** 1 College of Pharmacy and Research Institute of Pharmaceutical Science, Sookmyung Women’s University, Seoul, 140-742, Republic of Korea

## Abstract

**Background:**

In the present study, we investigated the changes of uptake and efflux transport of taurine under various stress conditions using rat conditionally immortalized syncytiotrophoblast cell line (TR-TBT cells), as *in vitro* blood-placental barrier (BPB) model.

**Methods:**

The transport of taurine in TR-TBT cells were characterized by cellular uptake study using radiolabeled taurine. The efflux of taurine was measured from the amount of radiolabeled taurine remaining in the cells after the uptake of radiolabeled taurine for 60 min.

**Results:**

Taurine uptake was significantly decreased by phosphorylation of protein kinase C (PKC) activator in TR-TBT cells. Also, calcium ion (Ca^2+^) was involved in taurine transport in TR-TBT cells. Taurine uptake was inhibited and efflux was enhanced under calcium free conditions in the cells. In addition, oxidative stress induced the change of taurine transport in TR-TBT cells, but the changes were different depending on the types of oxidative stress inducing agents. Tumor necrosis factor-α (TNF-α), lipopolysaccharide (LPS) and diethyl maleate (DEM) significantly increased taurine uptake, but H_2_O_2_ and nitric oxide (NO) donor decreased taurine uptake in the cells. Taurine efflux was down-regulated by TNF-α in TR-TBT cells.

**Conclusion:**

Taurine transport in TR-TBT cells were regulated diversely at extracellular Ca^2+^ level, PKC activator and oxidative stress conditions. It suggested that variable stresses affected the taurine supplies from maternal blood to fetus and taurine level of fetus.

## Introduction

Taurine, one of the essential nutrients, exists in high concentration in most tissues, where it shows various physiological functions such as including conjugation with bile acids, anti-oxidation, detoxification, osmoregulation, membrane stabilization and modulation of intracellular calcium level [1-4]. Also, taurine plays an important role in fetal development because taurine deficiency during pregnancy is associated with growth retardation, retinal degeneration and dysfunction of the central nervous system (CNS) [5,6]. In general, it was known that taurine can be supplied to most of human tissues by diet or biosynthesis in human body. However, although taurine is a pivotal amino acid at fetal and neonatal stages, the fetus has low capacities to synthesize taurine due to deficiency of enzyme, cysteine sulfinate decarboxylase [2]. Thus, taurine has to be supplied to the fetus from the maternal circulating blood via placenta.

The placenta regulates transport of nutrients and exchange of gases between maternal and fetal blood, and the blood-placenta barrier (BPB), which is composed of syncytiotrophoblast cells, has a key role in these functions [7]. It already has been reported that human placental syncytiotrophoblast possesses an active high-affinity transport system for taurine, the taurine transporter (TauT) [8,9]. TauT is dependent on sodium and chloride ion and is regulated by protein kinase C (PKC), glucose, hypertonicity and cytokine in various cells and organs such as brain, retina, intestinal cell and hepatic cell [2].

In the present study, we focused on the effect of oxidative stress on taurine transport in placenta. It was known that taurine plays a role as an antioxidant in the human body. Accordingly, transport activity of taurine may be also changed at the BPB under the pathophysiological conditions induced by oxidative stress, and this change could intensely affect the protective effect of taurine by influencing taurine concentration in the fetus. In the recent report, it showed that the level of taurine in the fetus at intrauterine growth restriction (IUGR) was low [10], NO level was also high in IUGR pregnancies [11]. It suggests a mutual relation between oxidative stress and taurine transport. However, the regulation of taurine transport at the BPB under oxidative stress remains uncertain at the present time. Thus, it is necessary to investigate the effect of oxidative stress on taurine transport in placenta. Oxidative stress in the cells is induced by increased oxidant generation, decreasing oxidant protection and failure of repairing oxidative damage. A reactive oxygen species (ROS) is generated *in vivo* by aerobic respiration, metabolism of xenobiotic compounds and inflammation induced by phagocytosis process [12]. Therefore, we evaluated the regulation of taurine transport under various conditions inducing oxidative stress such as pro-inflammatory cytokine, tumor necrosis factor-α (TNF-α); bacterial endotoxin, lipopolysaccharide (LPS); compound inducing a depletion of antioxidant such as glutathione, diethyl maleate (DEM); a ROS compound, hydrogen peroxide (H_2_O_2_); nitric oxide (NO) donor, 3-morpholinosyndomine (SIN-1).

Kitano *et al.* established a conditionally immortalized rat syncytiotrophoblast cell line, TR-TBT cells, from pregnant transgenic rat placenta at gestational day 18 [13]. TR-TBT 18d-1 and 18d-2 were originated from the syncytiotrophoblast I (maternal side) and II (fetal side), respectively, because rat syncytiotrophoblast consists of two layers while that of human is composed with one layer. TR-TBT cells is a good model for the analysis of the placental transport of nutrients [13,14]. We have carried out this study using TR-TBT cells as an *in vitro* BPB model.

## Materials and methods

### Materials

Radiolabeled [^3^H]taurine (SA 20.1 Ci/mmol) was obtained from NEN Life Science Products Inc. (Boston, MA). Tumor necrosis factor-α (TNF-α), lipopolysaccharide (LPS), 3-morpholinosyndomine (SIN-1), phorbol 12-myristate 13-acetate (PMA), nifedipine, nimodipine, verapamil, nickel chloride (NiCl_2_) and cadmium chloride (CdCl_2_) were purchased from Sigma Chemical (St. Louis, MO). Diethyl maleate (DEM) and hydrogen peroxide (H_2_O_2_) was obtained from Aldrich chemical Co. (St. Louis, MO) and Junsei chemical Co. (Tokyo, Japan), respectively.

### Cell culture

The TR-TBT cells were cultured with Dulbecco's modified Eagle's medium (Invitrogen, San Diego, CA), supplemented with 10% fetal bovine serum, 100 U/ml penicillin, and 100 μg/ml streptomycin (Invitrogen, San Diego, CA) at 33°C in a humidified atmosphere of 5% CO_2_/air. On rat tail collagen type I-coated 24 well culture plates (IWAKI, Tokyo, Japan) initial seeding was done at 1×10^5^ cells/well for the uptake study. After incubation for 3 days at 33°C, the cultures became confluent. Then the cells were incubated at 37°C for a further 3 days.

### Functional studies

The [^3^H]taurine uptake was performed according to the previous report [15]. TR-TBT cells were washed three times with 1 ml extracellular fluid (ECF) buffer consisting of 122 mM NaCl, 25 mM NaHCO_3_, 3 mM KCl, 1.4 mM CaCl_2_, 1.2 mM MgSO_4_, 0.4 mM K_2_HPO_4_, 10 mM D-glucose and 10 mM Hepes (pH 7.4) at 37°C. Uptake was initiated by applying 200 μL containing [^3^H]taurine (28 nM) at 37°C. Under a Ca^2+^ free condition, Ca^2+^ was replaced with Mg^2+^. [^3^H]Taurine uptake was stopped by removing the isotope ECF buffer and washing the cells with ice ECF buffer three times. To investigate the effect of phosphorylation of PKC on taurine uptake in TR-TBT cells, the cells was pre-incubated for 30 min in the presence of a PKC activator, PMA (1 μM). After that, [^3^H]taurine uptake study was performed as described above. To investigate the change of taurine uptake under the oxidative stress condition, the TR-TBT cells were pretreated with 20 ng/mL TNF-α, 10 ng/mL LPS, 100 μM DEM, 2 mM H_2_O_2_ or 1 mM SIN-1 for 3, 6, 9, 12 and 24 h, and the uptake study was performed as described above. The cells were then solubilized by incubation overnight in 750 µl of 1 N NaOH at RT. An aliquot (50 µl) was taken for quantification of cellular protein using a DC protein assay kit (Bio-Rad, Hercules, CA) with bovine serum albumin as a standard. The remaining solution (500 µl) was mixed with 5 ml of scintillation cocktail (Hionic-fluor; Packard, Meriden, CT) for measurement of radioactivity in a liquid scintillation counter (LS6500; Beckman, Fullerton, CA).

The efflux of [^3^H]taurine by TR-TBT cells was determined by incubating the cells for 60 min at 37°C with [^3^H]taurine dissolved in ECF buffer. The medium was then removed, and the cells were washed with ice-cold ECF buffer. The incubation medium alone or Ca^2+^ free medium was added for desired times. After appropriate time periods, the incubation medium was removed and the amount of [^3^H]taurine remained in the cells was measured. To induce oxidative stress in the cells, the TR-TBT cells were pretreated with 20 ng/mL TNF-α, 10 ng/mL LPS and 100 μM DEM for 9 hrs, 9 hrs and 12 hrs, respectively, and the efflux study was performed as described above.

### Data analysis

All data are given as mean ± SEM values. Statistical analyses were carried out by one-way ANOVA with Dunnett’s post-hoc test and p<0.05 was considered statistically significant.

## Results

### Effect of protein kinase C (PKC) on the taurine uptake in TR-TBT cells

**Figure 1:**
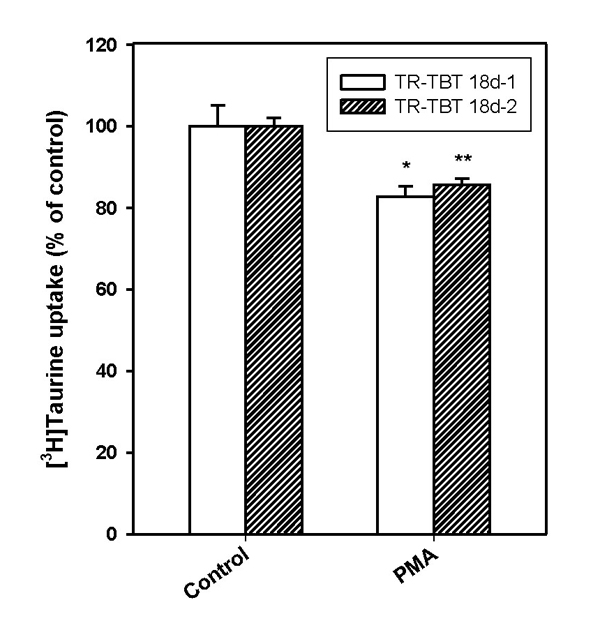
**Inhibition of [^3^H]taurine uptake by PKC activator in TR-TBT cells.** After 30 min pre-treatment of protein kinase C activator, phorbol 12-myristate 13-acetate (PMA), [^3^H]taurine (28 nM) uptake study was performed at 37°C for 5 min in TR-TBT 18d-1 cells (open bar) and TR-TBT 18d-2 cells (shaded bar). Each value represents the mean ± SEM (n=3). ^*^ p<0.05, ^**^ p<0.01 significantly different from control.

Taurine transport was regulated by phosphorylation of PKC in TR-TBT cells. As exposed to PKC activator, PMA (1 μM), [^3^H]taurine uptake was significantly decreased in TR-TBT cells (Fig. 1).

### Effect of Ca^2+^ and calcium channel blockers on the taurine transport in TR-TBT cells

**Figure 2:**
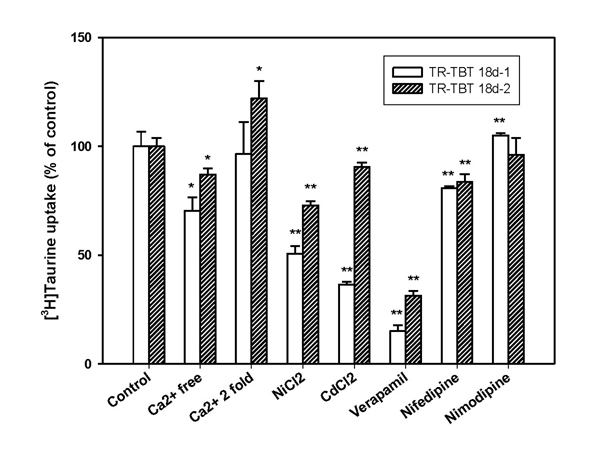
**Effect of Ca^2+^ and calcium channel blockers on [^3^H]taurine uptake by TR-TBT cells.** [^3^H]Taurine (28 nM) uptake was performed at 37°C for 5 min in ECF buffer in the absence or the presence of 2.8 mM calcium ion, 1 mM nickel chloride, 0.3 mM cadmium chloride, 0.3 mM verapamil, 2 mM nifedipine and 2 mM nimodipine in TR-TBT 18d-1 cells (open bar) and TR-TBT 18d-2 cells (shaded bar). Under Ca^2+^ free condition, Ca^2+^ was replaced Mg^2+^. Each value represents the mean ± SEM (n=3-4). ^*^ p<0.05, ^**^ p<0.01 significantly different control.

We investigated the effect of Ca^2+^ on taurine transport in TR-TBT cells by removal of Ca^2+^ from ECF buffer or addition of various calcium channel blockers in ECF buffer. Under Ca^2+^ free conditions, [^3^H]taurine uptake was significantly reduced in TR-TBT cells (Fig. 2). Various calcium channel blockers such as 0.3 mM verapamil, 2 mM nifedipine, 1 mM NiCl_2_ and 0.3 mM CdCl_2_ induced a significant decrease of the [^3^H]taurine uptake (Fig. 2). But, [^3^H]taurine uptake was not changed in the presence of nimodipine in the cells (Fig. 2). In the presence of 2.8 mM (2-folds) Ca^2+^, the [^3^H]taurine uptake had no change in TR-TBT 18d-1 cells, and was increased slightly in TR-TBT 18d-2 cells. The efflux of [^3^H]taurine was increased significantly in the cells under Ca^2+^ depletion condition (Table 1).

**Table 1:** Effect of calcium ion on [^3^H]taurine efflux in TR-TBT cells

Condition	Intracellular amount of [^3^H]taurine remained (% of time 0)	
	**TR-TBT 18d-1**	**TR-TBT 18d-2**

Control	100 ± 2	100 ± 3
Ca^2+^ free	72.1 ± 3.3 ^**^	75.7 ± 4.5 ^**^

Cells were incubated for 60 min at 37°C with ECF buffer containing [^3^H]taurine (28 nM). The cells were washed and then incubated with ECF buffer or Ca^2+^ free buffer for 30 min Under Ca^2+^ free condition, Ca^2+^ was replaced with Mg^2+^. Remaining of [^3^H]taurine in the cells was measured. Each value represents the mean ± SEM (n=3-4). ^**^ p<0.01 significantly different from normal efflux.

### Effect of oxidative stress on the taurine transport in TR-TBT cells

We investigated a change of the taurine uptake and efflux under oxidative stress conditions in TR-TBT cells. To induce various oxidative stress conditions, the cells were incubated in TNF-α (20 ng/mL), LPS (10 ng/mL), DEM (100 μM), H_2_O_2_ (2 mM) and SIN-1 (1 mM) for 3, 6, 9, 12 and 24 hrs. [^3^H]Taurine uptake was significantly increased by pre-treatment with TNF-α (20 ng/mL), LPS (10 ng/mL) and DEM (100 μM) for 24 hrs in the cells (Table 2). Whereas, as exposed to 2 mM H_2_O_2_ and 1 mM SIN-1 for 24 hrs in the cells, [^3^H]taurine uptake was significantly decreased (Table 2).

**Table 2:** Effect of oxidative stress inducing agents on [^3^H]taurine uptake in TR-TBT cells

Pretreatment	[^3^H]Taurine uptake (% of control)	
**Compounds**	**TR-TBT 18d-1**	**TR-TBT 18d-2**

Control	100 ± 7	100 ± 14
TNF- α 20 ng/mL	139 ± 11 ^*^	150 ± 12 ^**^
LPS 10 ng/mL	124 ± 4 ^**^	128 ± 7 ^*^
DEM 100 μM	137 ± 16 ^**^	154 ± 11 ^**^
H_2_O_2_ 2 mM	85.2 ± 1.8 ^**^	79.8 ± 3.2 ^**^
SIN-1 1 mM	66.8 ± 2.2 ^**^	84.2 ± 3.8 ^*^

Cells were treated with tumor necrosis factor-α (TNF- α, 20 ng/mL), lipopolysaccharide (LPS, 10 ng/mL), diethyl maleate (DEM, 100 μM), hydrogen peroxide (H_2_O_2,_ 2 mM) and 3-morpholinosyndomine (SIN-1, 1 mM) for 24 hrs, respectively. [^3^H]Taurine (28 nM) uptake was performed at 37°C for 5 min. Each value represents the mean ± SEM (n=3-4). ^*^p<0.05, ^**^ p<0.01 significantly different from the control.

**Figure 3:**
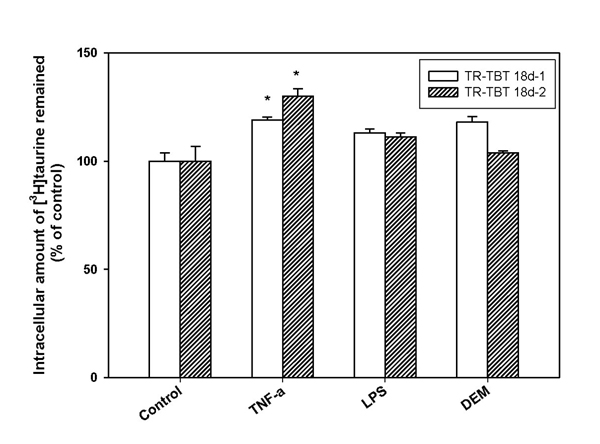
**Effect of oxidative stress inducing agents on [^3^H]taurine efflux by TR-TBT cells.** Cells were pre-treated with several stimulants for the specific time showed maximal taurine uptake, TNF-α (20 ng/mL) for 9 hrs, LPS (10 ng/mL) for 9 hrs and DEM (100 μM) for 12 hrs, respectively and then [^3^H]taurine efflux was examined in TR-TBT 18d-1 (open bar) cells and TR-TBT 18d-2 cells (shaded bar). Each value represents the mean ± SEM (n=3). ^*^ p < 0.05, significantly different from control.

 The effect of TNF-α, LPS and DEM pre-treatment on [^3^H]taurine efflux was also examined in TR-TBT cells. Fig. 3 showed the effect of TNF-α, LPS and DEM pre-treatment on the [^3^H]taurine efflux for the designated time. LPS and DEM pre-treatment did not affect [^3^H]taurine efflux significantly, but TNF-α treatment inhibited [^3^H]taurine efflux significantly in the cells (Fig. 3).

## Discussion

In the present study, we investigated the change of taurine transport via the placenta under various stress conditions using TR-TBT cells. It was reported that taurine transporter (TauT) existed in human placenta [8] and also the presence of TauT in TR-TBT cells was identified [14]. We also identified the characteristics of TauT which were previously known in various cells and organs such as brain, retina and intestinal cell [2] from the results (data not shown). The taurine uptake had time and sodium and chloride ion dependency and substrate specificity in TR-TBT cells. [^3^H]Taurine uptake was decreased significantly under extracellular calcium depletion (Fig. 2). The taurine uptake had calcium ion dependency as well as sodium and chloride ion, and it was sensitive to calcium depletion in extracellur fluid than calcium abundance (Fig. 2). In addition, the taurine efflux was evoked under calcium free condition about 20% over control (Table 1). These results suggested that an increase of taurine efflux might contribute to a decrease of taurine uptake at calcium free condition. Also, Na^+^-Ca^2+^ exchanger is associated with these effects of Ca^2+^ on taurine transport [4]. A change of Ca^2+^ concentration at intracellular or extracellular environment induced a change of Na^+^ concentration, and it may affect taurine transport by the cells because taurine transporter is very sensitive to Na^+^ concentration. Also, it was reported that Ca^2+^ free medium enhanced the taurine efflux in the rat striatum *in vivo *[17]. According to this study, the mechanism of an increase of taurine efflux by calcium depletion was considered that decreased calcium influx and increased nonspecific influx of Na^+^ into the cells induced an increase of taurine efflux [17]. Therefore, the decrease of taurine uptake under calcium depletion may be related with the increase of taurine efflux through the change of concentration of Na^+^ in the cells.

Taurine acts as an antioxidant, and has protection function when cell is damaged by oxidative stress [2]. This study showed that taurine uptake was increased under oxidative stress conditions for 24 hrs, TNF-α, LPS and DEM, respectively pro-inflammatory cytokine, bacterial endotoxin and a compound inducing oxidative stress by depleting of glutathione levels (Table 2). It was reported that mRNA level of TauT and taurine uptake by the cells was increased in brain capillary endothelial cells [18], astrocytes [19] and intestinal cells [16] by pre-treatment of TNF-α. The TNF-α signal is known to activate nuclear factor-κB (NF-κB) transcriptional activity by nuclear translocation [20]. Also, the NF-κB binding site is found in the TauT promoter region [20]. Therefore, it suggested that the TNF-α-NF-κB pathway could be associated in TauT up-regulation. DEM induced oxidative stress through the depletion of intracellular glutathione (GSH) [21] and up-regulated the cystine uptake in the organ [22]. Taurine is also sulfur-containing amino acid like cystine, therefore, it seems that DEM may induce an increase of taurine uptake in TR-TBT cells. It was reported that LPS reduced taurine uptake significantly in macrophage cell lines and Caco2 cells [23-25], had no effect in conditionally immortalized brain microvascular endothelial cells [18]. In macrophage cell line, it was explained that LPS decreased taurine uptake might be via the action of nitric oxide [23]. In the present study, LPS increased taurine uptake slightly in TR-TBT cells, and it is contrary to previous results. It is known that LPS stimulates production of many kinds of cytokines such as TNF-α. Therefore, it may be possible that taurine uptake was increased by TNF-α stimulated by LPS. Also, under hypertonic conditions, LPS activates taurine transport [24]. It is possible therefore to speculate that a switch controls the direction of LPS effect, but the complexity of the signaling pathways makes it very difficult [25]. The mechanism study of changes of taurine transport by LPS is needed to be further elucidated.

On the other hand, treatment of hydrogen peroxide induced reduction of taurine uptake (Table 2). Meng and colleagues showed that hydrogen peroxide elevated the phosphorylation of tyrosine residues via inhibition of tyrosine phosphatase [26]. Several of the serines, threonines and tyrosines in the intracellular domains of TauT are situated in motifs highly suitable as targets for protein kinases. In general, TauT is regulated by protein kinase C (PKC) phosphorylation of Ser-322, so activation of PKC inhibits taurine transport [2,3]. It was reported that there is possibility that a shift of TauT to a more tyrosine phosphorylated state in congruence with increased serine/threonine phosphorylation of TauT reduces the active taurine uptake following exposure to hydrogen peroxide [27]. We also identified that taurine uptake was inhibited by activator of PKC, PMA in TR-TBT cells (Fig. 1). It has been known that PMA activates Ca^2+^ dependent and Ca^2+^ independent PKCs [28]. Accordingly, an increased phosphorylation of TauT could be involved in H_2_O_2_-induced reduction of taurine uptake in TR-TBT cells.

Exposing the cells to nitric oxide donor induced a reduction of taurine uptake significantly in TR-TBT cells (Table 2). NO donor, SIN-1, releases NO, O_2_^-^ and ONOO^-^. It was demonstrated that ONOO^-^ interacted with TauT tyrosine residues and formation of nitrotyrosine was detected highly in NO donor treatment [29-32]. These alterations of transporter induced a reduction of taurine transport activity. In pregnant women having intrauterine growth restriction (IUGR) disease, placental transport capacity of taurine was reduced and fetal taurine level was decreased [33]. In addition, it has been previously shown that IUGR is associated with increased fetoplacental NO levels [12]. Thus, NO could play an important role in down-regulating TauT activity in IUGR.

Taurine efflux in TR-TBT cells was examined by measuring the remaining intracellular taurine. Our results showed that [^3^H]taurine efflux was significantly decreased by TNF-α pre-treatment, but had no change by LPS and DEM (Fig. 3). These results coincided with previous *in vivo* study in rat brain [34]. Thus, oxidative stress condition affected taurine efflux in TR-TBT cells as well as taurine uptake. Also, these results suggested that decrease of taurine efflux by TNF-α may be a possible mechanism for increase of taurine uptake to the cells.

## Conclusions

1. Taurine transport was regulated by PKC in TR-TBT cells. Also, Ca^2+^ was involved in taurine transport, and Ca^2+^ free conditions decreased taurine uptake and evoked taurine efflux.

2. Oxidative stress induced the change of taurine transport in TR-TBT cells, but the change was different as a type of oxidative stress inducing agents. Taurine uptake was increased by TNF-α, LPS and DEM pre-treatment, but decreased by H_2_O_2_ and NO stimulation. Whereas, taurine efflux was only regulated by TNF-α pre-treatment.

3. The taurine transport through the BPB was regulated in various stress conditions, and these results suggest that variable stresses affect the taurine supplies from maternal blood to fetus and taurine level of fetus.

## Competing interests

The authors declare that they have no competing interests.

## Authors’ contributions

Lee NY carried out the functional study, the experiments and helped to draft the manuscript. Kang YS participated in the data analysis and design of the study, and drafted the manuscript. All authors read and approved the final manuscript.

## References

[B1] Huxtable RJ Physiological actions of taurine. Physiol Rev 1992 72 101 163 1731369 10.1152/physrev.1992.72.1.101

[B2] Tappaz ML Taurine biosynthetic enzymes and taurine transporter: molecular identification and regulations. Neurochem Res 2004 29 83 96 10.1023/B:NERE.0000010436.44223.f8 14992266

[B3] Lambert IH Regulation of the cellular content of the organic osmolyte taurine in mammalian cells. Neurochem Res 2004 29 27 63 10.1023/B:NERE.0000010433.08577.96 14992263

[B4] Foos TM Wu JY The role of taurine in the central nervous system and the modulation of intracellular calcium homeostasis. Neurochem Res 2002 27 21 26 10.1023/A:1014890219513 11926272

[B5] Chapman GE Greenwood CE Taurine in nutrition and brain development. Nutr Res 1988 8 955 968 10.1016/S0271-5317(88)80135-0

[B6] Sturman JA Taurine in development. J Nutr 1988 118 1169 1176 3054019 10.1093/jn/118.10.1169

[B7] Jansson T Amino acid transporters in the human placenta. Pediatr Res 2001 49 141 147 10.1203/00006450-200102000-00003 11158505

[B8] Kulanthaivel P Cool DR Ramamoorthy S Mahesh VB Leibach FH Ganapathy V Transport of taurine and its regulation by protein kinase C in the JAR human placental choriocarcinoma cell line. Biochem J 1991 277 53 58 1854347 10.1042/bj2770053 PMC1151190

[B9] Ramamoorthy S Leibach FH Mahesh VB Han H Yang-Feng T Blakely RD Ganapathy V Functional characterization and chromosomal localization of a cloned taurine transporter from human placenta. Biochem J 1994 300 893 900 8010975 10.1042/bj3000893 PMC1138249

[B10] Norberg S Powell TL Jansson T Intrauterine growth restriction is associated with a reduced activity of placental taurine transporters. Pediatr Res 1998 44 233 238 10.1203/00006450-199808000-00016 9702920

[B11] Lyall F Greer I Young A Myatt L Nitric oxide concentrations are increased in the feto-placental circulation in intrauterine growth restriction. Placenta 1996 17 165 168 10.1016/S0143-4004(96)80009-9 8730886

[B12] Rubin E Essential Pathology. 2001 Maryland: Lippincott Williams&Wilkins

[B13] Kitano T Iizasa H Terasaki T Asashima T Matsunaga N Utoguchi N Watanabe Y Obinata M Ueda M Nakashima E Polarized glucose transporters and mRNA expression properties in newly developed rat syncytiotrophoblast cell lines, TR-TBTs. J Cell Physiol 2002 193 208 218 10.1002/jcp.10165 12384998

[B14] Kitano T Iizasa H Hwang IW Hirose Y Morita T Maeda T Nakashima E Conditionally immortalized syncytiotrophoblast cell lines as new tools for study of the blood-placenta barrier. Biol Pharm Bull 2004 27 753 759 10.1248/bpb.27.753 15187410

[B15] Lee NY Choi HM Kang YS Choline transport via choline transporter-like protein 1 in conditionally immortalized rat syncytiotrophoblast cell lines TR-TBT. Placenta 2009 30 368 374 10.1016/j.placenta.2009.01.011 19246089

[B16] Mochizuki T Satsu H Shimizu M Signaling pathways involved in tumor necrosis factor α-induced upregulation of the taurine transporter in Caco-2 cells. FEBS Letters 2005 579 3069 3074 10.1016/j.febslet.2005.04.063 15907840

[B17] Molchanova SM Oja SS Saransaari P Mechanisms of enhanced taurine release under Ca^2+^ depletion. Neurochem Int 2005 47 343 349 10.1016/j.neuint.2005.04.027 15982785

[B18] Kang YS Ohtsuki S Takanaga H Tomi M Hosoya KI Terasaki T Regulation of taurine transport at the blood-brain barrier by tumor necrosis factor-α, taurine and hypertonicity. J Neurochem 2002 83 1188 1195 10.1046/j.1471-4159.2002.01223.x 12437590

[B19] Chang RCC Stadlin A Tsang D Effects of tumor necrosis factor alpha on taurine uptake in cultured rat astrocytes. Neurochem Int 2001 38 249 254 10.1016/S0197-0186(00)00082-6 11099784

[B20] Han X Budreau AM Chesney RW Cloning and characterization of the promoter region of the rat taurine transporter (TauT) gene. Adv Exp Med Biol 2000 483 97 108 full_text 11787653 10.1007/0-306-46838-7_9

[B21] Bannai S Induction of cystine and glutamate transport activity in human fibroblast by diethyl maleate and other electrophilic agents. J Biol Chem 1984 259 2435 2440 6142042

[B22] Hosoya KI Saeki S Terasaki T Activation of carrier-mediated transport of L-cystine at the blood-brain and blood-retinal barriers in vivo. Microvasc Res 2001 62 136 142 10.1006/mvre.2001.2328 11516242

[B23] Kim HW Kim JH An HS Park KK Kim BK Park T Myo-inositol restores the inflammation-induced down-regulation of taurine transport by the murine macrophage cell line, RAW 264.7. Life Sci 2003 73 2477 2489 10.1016/S0024-3205(03)00656-8 12954456

[B24] Romio L Zegarra-Moran Varesio L Galietta LJV Regulation of taurine transport in murine macrophages. Amino Acids 2001 21 151 160 10.1007/s007260170022 11665811

[B25] O'Flaherty L Stapleton PP Redmond HP Bouchier-Hayes D Dexamethasone and lipopolysaccharide regulation of taurine transport in Caco-2 cells. J Surg Res 1997 69 331 336 10.1006/jsre.1997.5067 9224402

[B26] Meng TC Fukada T Tonks NK Reversible oxidation and inactivation of protein tyrosine phosphatases in vivo. Mol Cell 2002 9 387 399 10.1016/S1097-2765(02)00445-8 11864611

[B27] Voss JW Pedersen SF Christensen ST Lambert IH Regulation of the expression and subcellular localization of the taurine transporter TauT in mouse NIH3T3 fibroblasts. Eur J Biochem 2004 271 4646 4658 10.1111/j.1432-1033.2004.04420.x 15606752

[B28] Kazanietz MG Areces LB Bahador A Mischak H Goodnight J Mushinski JF Blumberg PM Characterization of ligand and substrate specificity for the calcium-dependent and calcium-independent protein kinase C isozymes. Mol Pharmacol 1993 44 298 307 8355667

[B29] Kulanthaivel P Leibach FH Mahesh VB Ganapathy V Tyrosine residues are essential for the activity of the human placental taurine transporter. Biochem Biophys Acta 1989 985 139 146 10.1016/0005-2736(89)90358-1 2804101

[B30] Myatt L Rosenfield RB Eis AL Brockman DE Greer I Lyall F Nitrotyrosine residues in placenta. Evidence of peroxynitrite formation and action. Hypertension 1996 28 488 493 8794838 10.1161/01.hyp.28.3.488

[B31] Roggensack AM Zhang Y Davidge ST Evidence for peroxynitrite formation in the vasculature of women with preclampsia. Hypertension 1999 33 83 89 9931086 10.1161/01.hyp.33.1.83

[B32] Khullar S Greenwood SL Mccord N Glazier JD Ayuk PTY Nitric oxide and superoxide impair human placental amino acid uptake and increase Na^+^ permeability: implications for fetal growth. Free Radic Biol Med 2004 36 271 277 10.1016/j.freeradbiomed.2003.11.007 15036346

[B33] Roos S Powell TL Jansson T Human placental taurine transporter in uncomplicated and IUGR pregnancies: cellular localization, protein expression, and regulation. Am J Physiol Regul Integr Comp Physiol 2004 287 R886 R893 15166008 10.1152/ajpregu.00232.2004

[B34] Lee NY Kang YS The brain-to-blood efflux transport of taurine and changes in the blood-brain barrier transport system by tumor necrosis factor-α. Brain Res 2004 1023 141 147 10.1016/j.brainres.2004.07.033 15364029

